# Gintonin stimulates dendritic growth in striatal neurons by activating Akt and CREB

**DOI:** 10.3389/fnmol.2022.1014497

**Published:** 2022-10-26

**Authors:** Hye Kyung Lim, Kitaek Kim, Youn Kyoung Son, Seung-Yeol Nah, Soo Min Ahn, Minseok Song

**Affiliations:** ^1^Department of Life Sciences, Yeungnam University, Gyeongsan, South Korea; ^2^National Institute of Biological Resources, Incheon, South Korea; ^3^Ginsentology Research Laboratory and Department of Physiology, College of Veterinary Medicine, Konkuk University, Seoul, South Korea; ^4^Department of Pediatric Surgery, Metabolic and Bariatric Surgery Center, Gangnam Severance Hospital, Yonsei University College of Medicine, Seoul, South Korea

**Keywords:** gintonin, dendritic growth, LPA, BDNF, striatal neurons

## Abstract

Gintonin, a glycolipid protein conjugated with lysophosphatidic acid (LPA), is a newly identified compound extracted from Korean ginseng. LPA receptor isotypes exhibit high affinity for gintonin and mediate intracellular calcium signaling in various animal cell models. In this study, we found that gintonin induced the activation of Akt and cAMP-response element binding protein (CREB) in mouse striatal neurons, and chronic treatment with gintonin potently induced dendritic growth and filopodia formation. Gintonin-induced Akt/CREB activation and dendritic development were significantly impaired by LPA receptor (LPAR1/3) inhibition with Ki16425. Intriguingly, prolonged treatment with gintonin ameliorated the reduction in dendritic formation caused by Shank3 and Slitrk5 deficiency in the striatal neurons. In addition, gintonin and brain-derived neurotrophic factor (BDNF) had a synergistic effect on AKT/CREB activation and dendritic growth at suboptimal concentrations. These findings imply that gintonin-stimulated LPA receptors play a role in dendritic growth in striatal neurons and that they may act synergistically with BDNF, which is known to play a role in dendritogenesis.

## Introduction

Gintonin, a glycolipid protein conjugated with lysophosphatidic acids (LPA), is a newly identified compound extracted from Korean ginseng (Hwang et al., 2012b; Choi et al., 2015). Administration of gintonin resulted in transient increases in intracellular calcium concentration in various animal cell models, including cultured hippocampal neurons, and elevated long-term potentiation (LTP) in rat hippocampal slices (Shin et al., 2012; Hwang et al., 2012b, 2015). Several studies have shown that gintonin stimulates the LPA receptor and subsequently activates both the phosphoinositide 3-kinase (PI3K) pathway and the phospholipase C (PLC)/inositol 1,4,5-triphosphate (IP3)/protein kinase C (PKC) pathway (Shin et al., 2012; Hwang et al., 2012a,b). Gintonin-induced LPA receptor activation led to the enhancement of synaptic vesicle release and potentiated both inhibitory and excitatory neurotransmission in hippocampal synapses (Park et al., 2015). Gintonin stimulated the LPA receptor/PLC/IP3 signaling pathway to induce dopamine release in PC12 cells (Hwang et al., 2015). Furthermore, gintonin affected neuronal proliferation, migration, and morphological changes in the adult mouse brain (Kim et al., 2021).

LPA is a lipid molecule present in various tissues and fluids and is known to act as a ligand for G-protein-coupled receptors (GPCR) called LPA receptors that are located in the cell membrane (Trimbuch et al., 2009; Suckau et al., 2019). LPA receptor signaling is known to be involved in intracellular calcium signaling in a variety of cells and is particularly involved in the development of neural progenitor cells, synaptic transmission, growth cone collapse, neurite retraction, post-mitotic neuronal migration, and immune response in the brain (Schmidt et al., 1999; Fukushima et al., 2000, 2002; Campbell and Holt, 2001; Yuan et al., 2003; Segura et al., 2004). Several studies have suggested that LPA2 and LPA3 receptors are involved in neurite branching and elongation in the hippocampus neurons (Furuta et al., 2012; Uenaka et al., 2022). LPA stimulated the LPA1 receptor and initiated LPA signaling, which induced extracellular signal-regulated kinase (ERK)1/2 and cAMP-response element binding protein (CREB) phosphorylation in mouse hippocampal neurons (Olianas et al., 2017). Loss of LPA signaling caused by genetic ablation of lysophosphatidic acid receptor 1 (LPAR1) led to defects in neural progenitor cell (NPC) proliferation and self-renewal (Hu et al., 2021). Genetic research and pharmacological studies have revealed that LPAR1 is essential for regulating emotions and mood (Castilla-Ortega et al., 2011; Garcia-Fernandez et al., 2012; Pedraza et al., 2014).

Neurons primarily produce structurally polarized morphologies, which in turn provide the foundation for complicated and extensive networks of the nervous system to ensure reliable brain functioning. Dendrites are a distinguishing feature of polarized neuronal structures that receive nerve impulses from neighboring neurons in the network. For the brain to develop, the dendritic arbor should acquire the appropriate morphology, which leads to optimal connectivity in neuronal circuits. This process, which is very tightly controlled developmentally, can only be accomplished by organic communication between numerous extrinsic factors and intrinsic components that respond to them (Jan and Jan, 2003; Urbanska et al., 2008; Jan and Jan, 2010). Numerous stimuli that control axon outgrowth and guidance have been discovered; however, the molecules that regulate dendritic outgrowth remain mostly unknown. Dendritic outgrowth and stabilization are influenced by extrinsic stimuli such as BDNF and its receptor TrkB (Horch and Katz, 2002; Gorski et al., 2003). Semaphorin 3A has chemorepellant properties in the axon and chemoattractive properties in the dendrites of cortical pyramidal neurons (Polleux et al., 2000). Dendrite development and branching have been reported to be impacted by ligands such as Wnt, transforming growth factor beta (TGFb), glial cell line-derived neurotrophic factor (GDNF), and fibroblast growth factor (FGF); however, further investigation is necessary (Kirszenblat et al., 2011; Ting et al., 2014; Irala et al., 2016; Huang et al., 2017). It is vital to recognize external stimuli and comprehend how they interact in a manner that encourages or restricts dendrite growth and branching in neurons.

In this study, we found that prolonged treatment with gintonin potently induced dendritic growth *via* Akt and CREB activation in mouse striatal neurons. Additionally, gintonin enhanced the density of dendritic spines, particularly those of filopodia. Gintonin-induced Akt/CREB activation and dendritic growth are dependent on LPA receptor activation. Intriguingly, gintonin was able to stimulate dendritogenesis even when dendrite growth was hindered by knockdown of Shank3 and slitrk5, which are linked to autism and obsessive–compulsive disorder, respectively. Additionally, gintonin and BDNF exhibited a synergistic effect on Akt activation and dendritic growth at suboptimal dosages. These findings demonstrate that even in striatal neurons where growth is impaired by genetic defects, gintonin may stimulate dendritic growth, which can enhance the impact of BDNF.

## Materials and methods

### Reagents and antibodies

Human recombinant BDNF was purchased from PeproTech (Rocky Hill, NJ, United States). Rabbit anti-Akt and rabbit anti-phospho-AktS473 were obtained from Cell Signaling Technology (Danvers, MA, United States). Chicken anti-microtubule-associated protein 2 (MAP2) and rabbit anti-hemagglutinin (HA) were obtained from Abcam (Cambridge, MA, United States). Alexa Fluor® dye-conjugated secondary antibodies were obtained from Invitrogen (Carlsbad, CA, United States). Horseradish peroxidase (HRP)-conjugated goat anti-mouse or rabbit IgG and HRP-conjugated rabbit anti-goat IgG were purchased from Calbiochem (La Jolla, CA, United States). Recombinant anti-CREB (phospho S133) antibody [E113] (ab32096), anti-CREB antibody (ab31387), and mouse anti-GFP were obtained from Abcam (Boston, MA, United States). Ki16425 was purchased from Sigma-Aldrich. All cell culture reagents were purchased from Invitrogen and all the other reagents were purchased from Sigma-Aldrich.

### Primary neuronal culture

E17 mice embryo was obtained from pregnant mice. The brain was separated from the skull, and the cortical and striatal tissues were then removed and kept in dissection media (1x Hanks’ balanced salt solution (HBSS), 0.4% glucose). Trypsin-ethylenediaminetetraacetic acid (EDTA; 0.5%) was diluted to a final concentration of 0.05% in DM containing the tissue and incubated for 15 min at 37°C. Following trypsinization, the tissue was placed in a 15 ml tube and treated with plating media (Minimum essential medium (MEM), 10% fetal bovine serum (FBS), sodium pyruvate, and Primocin) to remove the remaining trypsin. PM was added to disrupt the cells after the tissue and supernatant were separated by centrifugation for 5 min at 800 rpm. Striatal and cortical neurons were plated on polyD lysine/laminin-coated glass coverslips at a density of 1.5 × 10^4^ cells/cm^2^ for immunocytochemistry or on polyD lysine/laminin-coated culture dishes at a density of 1 × 10^5^ cells/cm^2^ for biochemical experiments. The PM was removed on day *in vitro* (DIV)2, and replaced with complete medium (CM; Neurobasal, B-27, L-Glutamine, and Primocin); CM containing AraC was added on DIV4. Every 2–3 days, half of the medium in which the cells were growing was replaced with new CM. To examine the activation of Akt/CREB, the CM was replaced with a starvation medium (Neurobasal, 0.2% glucose) for at least 12 h before treatment with gintonin.

### Plasmid constructs and siRNA

The pLVTHM vectors (Addene #12247; Wiznerowicz and Trono, 2003) were used to transcribe functional small interfering RNA (siRNA) of mice Shank3 and Slitrk5. In the vectors, oligonucleotides targeting different genes were inserted into the downstream of H1 promoter, with their veracity confirmed by double digestion and sequencing. The target sequences for Slitrk5 and Shank3 are, respectively, 5′-GCAGAAACCATCGATTATT-′3 GCATTCAACCAGAGCTCAGAT, and 5′-CCACGTCACTCACAAGTTTCT-′3. The expression levels of Slitrk5 and Shank3 in mouse cortical neurons transfected with the resulting siRNA or the scramble siRNA were analyzed by qRT-PCR and Western blot.

### Calcium phosphate transfection

Before transfection, the CM was transferred to a 15 ml tube, and neurons were incubated with MEM. The DNA solution was mixed with 2 × HBS and incubated at room temperature for 20 min. After incubation, the DNA-HBS mixture was added dropwise to the neurons. Neurons were incubated for 30 min at 37°C. The calcium phosphate-DNA complex was removed, and the neurons were then incubated with the collected CM at 37°C.

### Western blot

NP-40 lysis buffer (1% Nonidet P-40 substitute 40, 150 mM NaCl, 20 mM Tris–HCl pH 7.4, and 1 mM EDTA) containing protease and phosphatase inhibitors (2 g/ml leupeptin, 2 g/ml aprotinin, 1 mM sodium orthovanadate, 10 mM sodium fluoride, and 1 mM phenylmethylsulfonyl fluoride) was used to lyse the neuronal cultures. The Lowry assay was used to measure the amount of protein in the lysates after they were cleared by centrifugation at 12,000 × *g* for 15 min (DC protein assay kit, Bio-Rad). The protein samples were heated for 5 min in lithium dodecyl sulfate (LDS) sample buffer (NuPAGE Novex, Invitrogen, Carlsbad, CA, United States) before it was separated on a 10% NuPAGE Bis-Tris gel, and transferred to polyvinylidene difluoride (PVDF) membranes. They were then blocked for an hour in Tris-buffered saline (TBS) with 0.1% Tween 20 and 5% low-fat milk (TBS-T). Primary antibodies were incubated overnight at 4°C in TBST containing 3% bovine serum albumin (BSA). After washing in TBST and incubating with horseradish peroxidase (HRP) secondary antibodies at room temperature for an hour, immunoreactive proteins were detected using the ECL™ Prime western blotting System/GE Healthcare/ RPN2232 treatment and Vilber Fusion Solo X. The membranes were first washed in 0.1 M glycine pH 2.5 for 15 min and then in 1% sodium dodecyl sulfate (SDS) for another 15 min to complete the stripping procedure. Each experiment was conducted at least three times. Western blotting was performed by subtracting the background value from each quantification using ImageJ software. The ratio value is expressed as a graph using Prism 6.

### Immunocytochemistry

Serum-starved DIV7–9 mouse striatal neurons were provided with 2% glucose overnight in Neurobasal medium. For 15 min at room temperature (RT), the cells were fixed in 3.7% formaldehyde solution (EMS). Unreacted formaldehyde was quenched with glycine. With mild agitation at RT for 6 min, 0.2% Triton-X was used for permeabilization. Primary antibodies [Anti-MAP2 antibody (ab5392)] were incubated for 60 min at room temperature before being gently shaken while being washed in PBS. Alexa-conjugated secondary antibodies were added and incubated for 20 min at room temperature. The antibodies were prepared in a blocking solution of PBS, 10% donkey serum, and 3% BSA.

### Dendrite and spine analysis

Not less than 30 cells were analyzed for each sample. A LEICA DMi8 microscope and an ANDOR Zyla sCMOS camera were used to capture cell images. LAS X software was used to acquire the images, and ImageJ software was used to analyze the data. Direct counting by eye was performed for both the primary and secondary dendrite counts. Prism 6 shows the number of dendrites in each sample. Images for dendritic spine analysis were obtained using a Nikon A1si confocal microscope. The number of dendritic spines per 10 μm was investigated using ImageJ after maximal projection following a 0.2 um interval Z-series shoot. Based on their morphological characteristics, dendritic spines are divided into 4 groups. The head diameter of mushroom spines is larger than the neck diameter, whereas the head diameter of thin spines is the same as the neck diameter. Spines are longer than the diameter of the thorn, while filopodia are longer than the diameter, and blunt thorns have a diameter that is either equal to or less than the length of the thorn. Dendrite length measurement and Sholl analysis were performed using ImageJ software.

## Results

### Gintonin activates Akt/CREB signaling in primary cortical neurons

**Figure 1:**
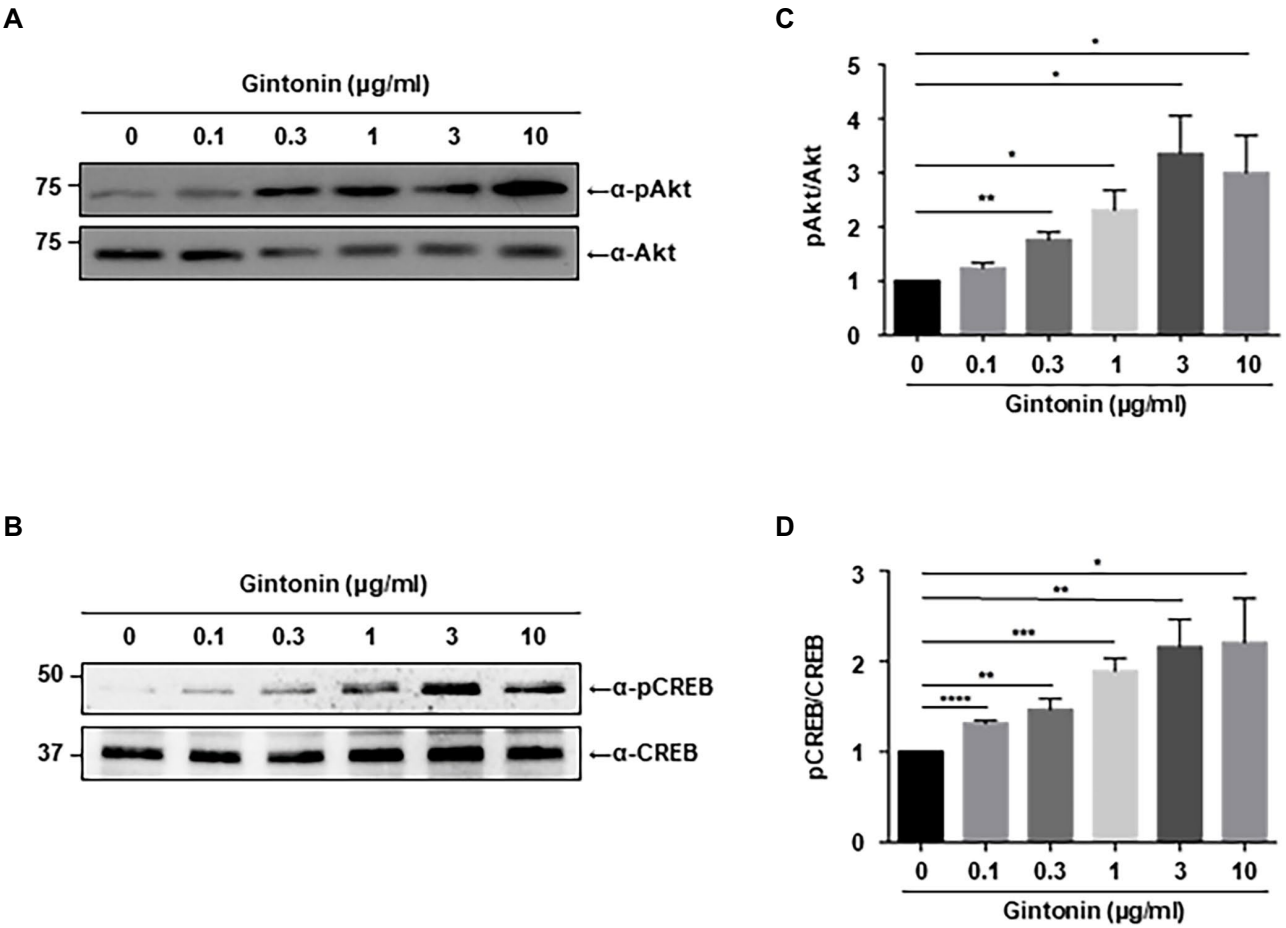
Gintonin induces Akt/CREB phosphorylation in the cortical neurons. **(A,B)** Representative blots showing the effect of gintonin on Akt and CREB phosphorylation. Primary cortical neurons (DIV7) were treated with the indicated concentration of gintonin for 2 h, and the lysates were then used for immunoblot analysis of phospho-Akt, Akt, phospho-CREB, and CREB. Scale bar, 2 μm. **(C,D)** Densitometric quantification of the results shown on the right. Results are means ± standard error of mean (SEM) from three independent experiments (*p < 0.05, **p < 0.01, ***p < 0.0005, ****p < 0.0001, Student’s *t*-test).

Chronic activation of PI3K-Akt signaling plays a pivotal role in dendritic elongation and growth (Schmelzle and Hall, 2000). Our search for compounds that stimulate dendritic growth in neurons showed that gintonin stimulates Akt, which is essential for dendritic formation. We first assessed the phosphorylation of Akt in primary cortical neurons. DIV7 mouse cortical neurons were serum-starved overnight and treated with the indicated dose of gintonin for 2 h. Gintonin treatment at 0.1 μg/ml exhibited no discernible effect on the phosphorylation of Akt. Gintonin did, however, phosphorylate Akt in a dose-dependent manner from 0.3 to 3 μg/ml, after which the activation of Akt reached saturation (Figure 1A). This suggests that gintonin is sufficient to enhance Akt signaling in cortical neurons.

Next, we examined CREB signaling, which is known to stimulate dendritic arborization and synaptic plasticity in neurons (Redmond et al., 2002; Wayman et al., 2006; Kim et al., 2016). Various signaling pathways lead to the phosphorylation and activation of CREB, and CREB-mediated transcriptional regulation is essential for dendritic arborization (Lonze and Ginty, 2002; Wang et al., 2018). Dendritic growth triggered by BDNF/TrkB signaling is also mediated by CREB activation (Gonzalez et al., 2019). Immunoblot analysis was performed to examine the effect of gintonin on CREB phosphorylation in primary cortical neurons. The results showed a dose-dependent increase in CREB phosphorylation from 0.3 to 3 μg/ml of gintonin, similar to that of gintonin-induced Akt phosphorylation (Figure 1B).

### Gintonin stimulates dendrite outgrowth in the primary striatal neurons

**Figure 2:**
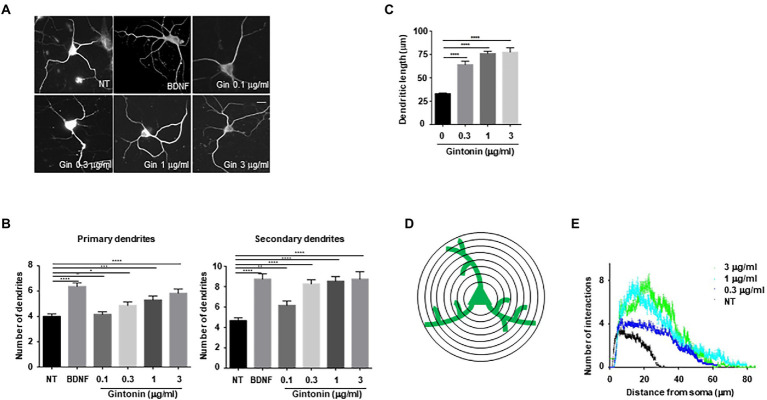
Gintonin induces dendritic growth in striatal neurons. Cultured striatal neurons were treated with the indicated dose of gintonin at DIV2. After 5 d of exposure, the cultures were fixed and stained with anti-microtubule-associated protein 2 (MAP2) antibody. Neuronal processes were counted with fluorescence microscopy. BDNF (40 ng/ml) was used as a positive control for striatal dendritic growth. **(A)** Representative images of gintonin and BDNF-treated striatal neurons. **(B)** Quantitation of the number of primary and secondary dendrites in gintonin-treated striatal neurons. **(C)** The total length of dendrites for gintonin and BDNF-treated neurons. **(D, E)** Sholl analysis of the dendritic arbor from gintonin-treated striatal neurons. Results are presented as means ± SEM from three independent experiments determined from the analysis of 40 neurons per condition per experiment (*p < 0.05, **p < 0.01, ***p < 0.0005, ****p < 0.0001, Student’s *t*-test).

To examine whether increased phosphorylation of Akt and CREB affects dendritic growth in primary neurons, an earlier experimental approach that demonstrated the impact of BDNF on dendritic growth in striatal neurons was employed (Baquet et al., 2004; Song et al., 2015). A previous study found that treating cultured striatal neurons with BDNF for 5 days resulted in a considerable increase in the number and length of dendrites. Using BDNF as a positive control, primary striatal neurons were treated with the indicated concentrations of gintonin for 5 days. Gintonin administration caused dendritic development in striatal neurons, and this effect was dose-dependent. After prolonged exposure to 3 μg/ml of gintonin, primary striatal neurons displayed a 1.5-fold (5.8 ± 0.37) higher number of primary dendrites and a 2-fold (9.08 ± 0.54) higher number of secondary dendrites compared to that of nontreated neurons (Figures 2A,B). Considering that BDNF is one of the molecules that most significantly induces dendritic growth in neurons, it is interesting to note that gintonin had a comparable effect on BDNF at a concentration of 3 μg/ml. Gintonin treatment at low doses (0.1 and 0.3 μg/ml) did not result in a significant increase in primary dendrites, but the secondary dendrites exhibited a considerable increase. This raises the possibility that gintonin-induced stimulation may have additional effects on secondary dendritic growth. Further, the length of the dendrites was measured, which displayed the effect of gintonin on striatal neurons (Figure 2C). Conventional Sholl analysis demonstrated that chronic exposure to gintonin increased the number of interactions between dendrites and the cell body of neurons (Figures 2D,E), which is consistent with earlier results. Even at a concentration of 10 μg/ml for 2 h, gintonin successfully activated Akt and CREB2; however, a longer exposure for 5 days resulted in cell death in striatal neurons. These results confirm that prolonged gintonin exposure stimulates dendritic outgrowth in mouse striatal neurons.

### Gintonin affects the filopodia-type of dendritic spine formation

**Figure 3:**
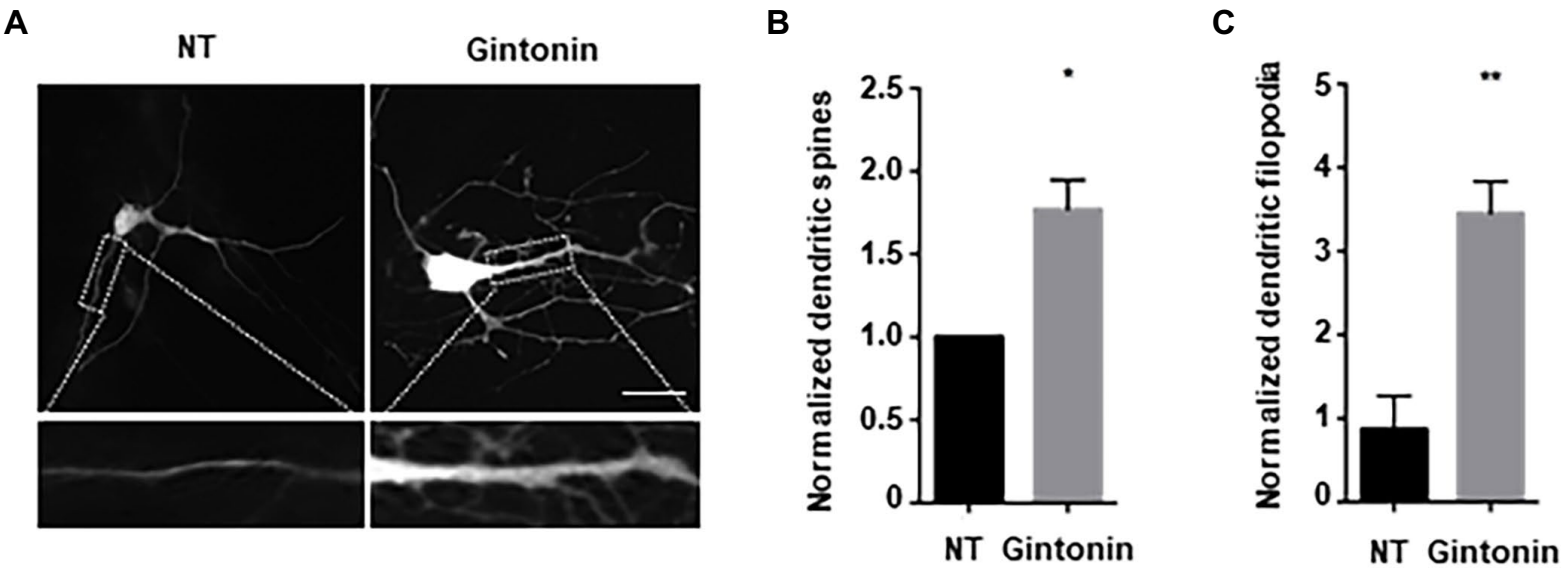
Gintonin increases filopodia-type dendritic spine density. **(A)** Representative images showing dendritic spine formation upon gintonin treatment in striatal neurons. DIV9 striatal neurons were treated with 3 μg/ml of gintonin for 5 days, until DIV14. Striatal neurons were transfected with GFP vector at DIV12 to visualize dendritic spines. Neurons were fixed at DIV14, and spines were counted in maximum-intensity projections of the z-stacks using ImageJ software. Scale bar, 2 μm Quantification of total **(B)** and filopodia-type spines **(C)**. Results are presented as means ± SEM from three independent experiments determined from the analysis of 40 neurons per condition per experiment (*p < 0.05, **p < 0.01, Student’s *t*-test).

Next, we examined the effect of gintonin on the morphological development of other neurons. In the early stages of neural development, dendritic shafts are heavily occupied by dendritic filopodia, which gradually develop into dendritic spines (Takahashi et al., 2003). Dendritic filopodia may be the putative sites of synapses, playing a significant role in structural plasticity along with dendritic spines. Gintonin was administered to primary striatal neurons at DIV9 for 5 days to determine whether it had any impact on the growth of dendritic spines. Neurons were transfected with green fluorescent protein (GFP) plasmids at DIV12 to visualize the dendritic spines. After 48 h post-transfection, neurons were fixed and analyzed for dendritic spine formation. The quantification of dendritic spines was approximately 2-fold higher in treated than that in nontreated neurons (Figures 3A,B). According to their size and shape, dendritic spines can be categorized as filopodia, stubby, thin, or mushroom spines (Peters and Kaiserman-Abramof, 1970). Gintonin was examined to determine whether it enhanced particular spine types, and surprisingly, gintonin-treated striatal neurons had 3 times higher amount of filopodia compared to that in nontreated controls, whereas the mushroom type was rarely observed (Figures 3A,C). These results suggest that gintonin can induce dendrite growth and the formation of filopodia structures, which can act as a foundation for the development of dendritic spines.

### LPAR1/3 inhibition impaired gintonin-induced Akt/CREB activation and dendritic growth

**Figure 4:**
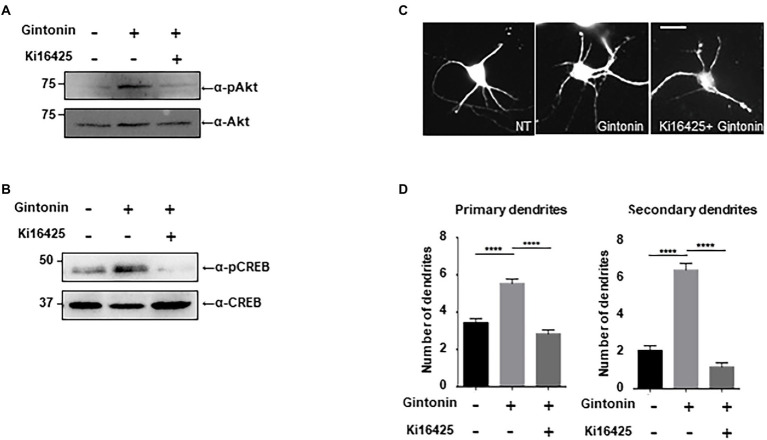
Gintonin-induced Akt/CREB activation and dendritic growth are abolished by LPAR inhibition with Ki16425. **(A,B)** Representative blots showing the effect of Ki16525 on gintonin-induced Akt and CREB phosphorylation. DIV7 cortical neurons were pretreated with 1uM Ki16425 for 1 h before administration of 3 μg/ml gintonin for 2 h. Lysates were used for immunoblot analysis of phospho-Akt, Akt, phospho-CREB, and CREB. **(C)** Representative images of gintonin-treated and Ki16525-cotreated striatal neurons. Cultured striatal neurons were treated with 3 μg/ml of gintonin in the presence and absence of 0.5 uM Ki16425 at DIV2. After 5 days of exposure, the cultures were fixed and stained with anti-MAP2 antibody. Neuronal processes were counted with fluorescent microscopy. Scale bar, 2 μm **(D)** Quantitation of the number of primary and secondary dendrites is shown in **(C)**. Results are presented as means ± SEM from three independent experiments determined from the analysis of 40 neurons per condition per experiment (***p < 0.0005, ****p < 0.0001, Student’s *t*-test).

Gintonin is an LPA and protein conjugate that elevates intracellular calcium in a target cell by activating LPA receptors (Shin et al., 2012; Hwang et al., 2012a,b). LPA receptors are heterogeneously expressed in the lungs, heart, ovaries, testes, stomach, and brain at relatively high concentrations (Noguchi et al., 2009; Choi et al., 2010). LPA1 is restrictively expressed in the proliferative cortical VZ of the embryonic brain (Choi et al., 2010). LPA2 and LPA3 are also expressed in the developmental brain, but their expression declines a week after birth (Contos et al., 2000). We determined whether the activation of Akt/CREB and dendritic growth by gintonin was achieved through activation of the LPA receptor. Following ligand interaction with LPA receptor types 1, 2, 3, 4, and 6, Akt/CREB can be activated *via* Gαi and phosphoinositide 3-kinases (PI3K)/ phosphoinositide-dependent kinase 1 (PDK1; Geraldo et al., 2021). Previous studies have shown that Ki16425, an antagonist of the LPA1/LPA3 receptor, abolishes the effects of gintonin (Ohta et al., 2003; Shin et al., 2012). KI16425 was used to determine whether LPA1/3 was involved in gintonin-induced Akt/CREB activation and dendritic growth. DIV7 cortical neurons were pretreated with 1 μM Ki14625 and exposed to 3 μg/ml of gintonin after 1 h. Gintonin treatment potently induced Akt and CREB phosphorylation. However, pretreatment with Ki14625 almost completely abolished gintonin-induced Akt and CREB phosphorylation (Figure 4A). Therefore, gintonin activates Akt and CREB phosphorylation by binding to the LPA1/3 receptors.

Next, we examined how gintonin-induced dendritic growth is altered in the presence of Ki16425. DIV2 striatal neurons were maintained with 3 μg/ml gintonin for 5 days in the presence or absence of Ki16425. Ki16425 treatment completely blocked the increase in primary and secondary dendrite numbers induced by prolonged gintonin treatment (Figures 4B–D). These findings demonstrate that gintonin facilitates dendritic development by activating the LPA1/3 receptors. Therefore, the phosphorylation of Akt and CREB by gintonin stimulates dendritic outgrowth through the LPA1/LPA3 receptors in striatal neurons.

### Gintonin rescues abnormal dendritic growth in Slitrk5 and Shank3 knockdown primary striatal neurons

In neuropsychiatric illnesses such as autism spectrum disorder (ASD) and obsessive–compulsive disorder (OCD), there is a strong correlation between the deficiency in dendritic development, and synaptic plasticity (Forrest et al., 2018). Gintonin enhances dendritic growth and spine formation; therefore, we aimed to determine whether it can be used as a remedy for neuropsychiatric conditions. In this study, we used defects in the Slitrk5 and Shank3 genes, which are typical of the genetic etiology of ASD and OCD, respectively, to evaluate the beneficial effects of gintonin on striatal dendritic growth. A transmembrane protein called Slitrk5 is present in the postsynaptic membrane and functions as an adhesion molecule that binds to the synaptic proteins to establish synapses. By facilitating the recycling of TrkB, Slitrk5 is involved in BDNF signaling and is also known to be associated with OCD (Song et al., 2015). A major scaffold protein, Shank3, is localized in the dendrites of postsynaptic neurons. Shank3 participates in the development and maintenance of synapses and is associated with the cytoskeleton and various postsynaptic proteins. Shank3 deletion results in abnormal dendritic spine formation and ASD in both mice and humans (Durand et al., 2012).

**Figure 5:**
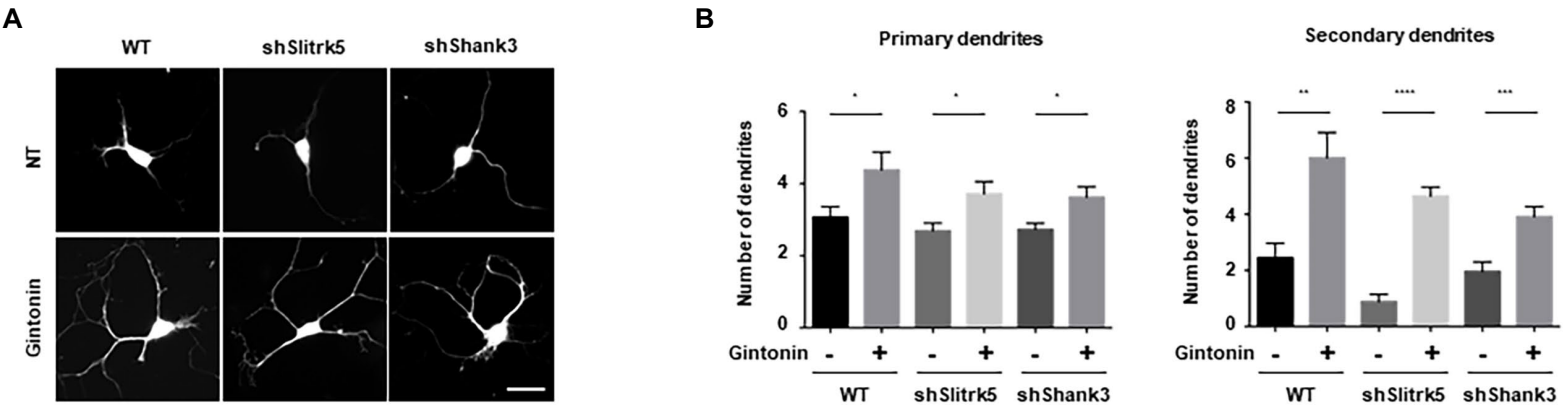
Gintonin rescued the abnormal dendritic growth in striatal neurons with Slitrk5 and Shank3 knockdown. **(A)** Representative images of gintonin-treated striatal neurons with wild-type (WT), Slitrk5, and Shank3 knockdown. DIV2 striatal neurons were transfected with pLVTHM scrambled, pLVTHM-shSlitrk5, and pLVTHM-shShank3, and treated with 3 μg/ml of gintonin for 5 d. The cultures were fixed and stained with anti-MAP2 antibody. Neuronal processes were counted with fluorescent microscopy. Scale bar, 2 μm. **(B)** Quantitation of the number of primary and secondary dendrites is shown. Results are presented as means ± SEM from three independent experiments determined from the analysis of 40 neurons per condition per experiment (*p < 0.05, **p < 0.01, ***p < 0.0005, ****p < 0.0001, Student’s *t*-test).

DIV2 striatal neurons were transfected with an empty pLVTHM vector, pLVTHM-Slitrk5 shRNA, and pLVTHM-Shank3 shRNA to knock down the expression of Slitrk5 and Shank3. Following gintonin treatment for 5 d after transfection, the images of shRNA-transfected striatal neurons were obtained using the GFP signal of the pLVTHM vector. Only a slight decrease in the number of primary dendrites was observed, and the expression of shRNAs had no discernible impact on the basal level. However, knocking down the Slitrk5 gene lowered the secondary dendrites of striatal neurons by less than 50%, while knocking down the Shank3 gene had no discernible impact. Gintonin administration for a prolonged period of time increased dendritic development in wild-type neurons as well as Slitrk5 and Shank3 knockdown neurons (Figures 5A,B). Gintonin treatment partially rescued the defective dendritic growth caused by Slitrk5 and Shank3 knockdown in primary and secondary dendrites by >60% and approximately 80%, respectively. Gintonin can restore dendritic development despite reduced BDNF signaling and Shank3 activity, which is an intriguing finding. These results suggest that gintonin may be a useful treatment for patients whose dendritic development is hindered by genetic predisposition.

### Gintonin and BDNF have a synergistic effect on dendritic growth

**Figure 6:**
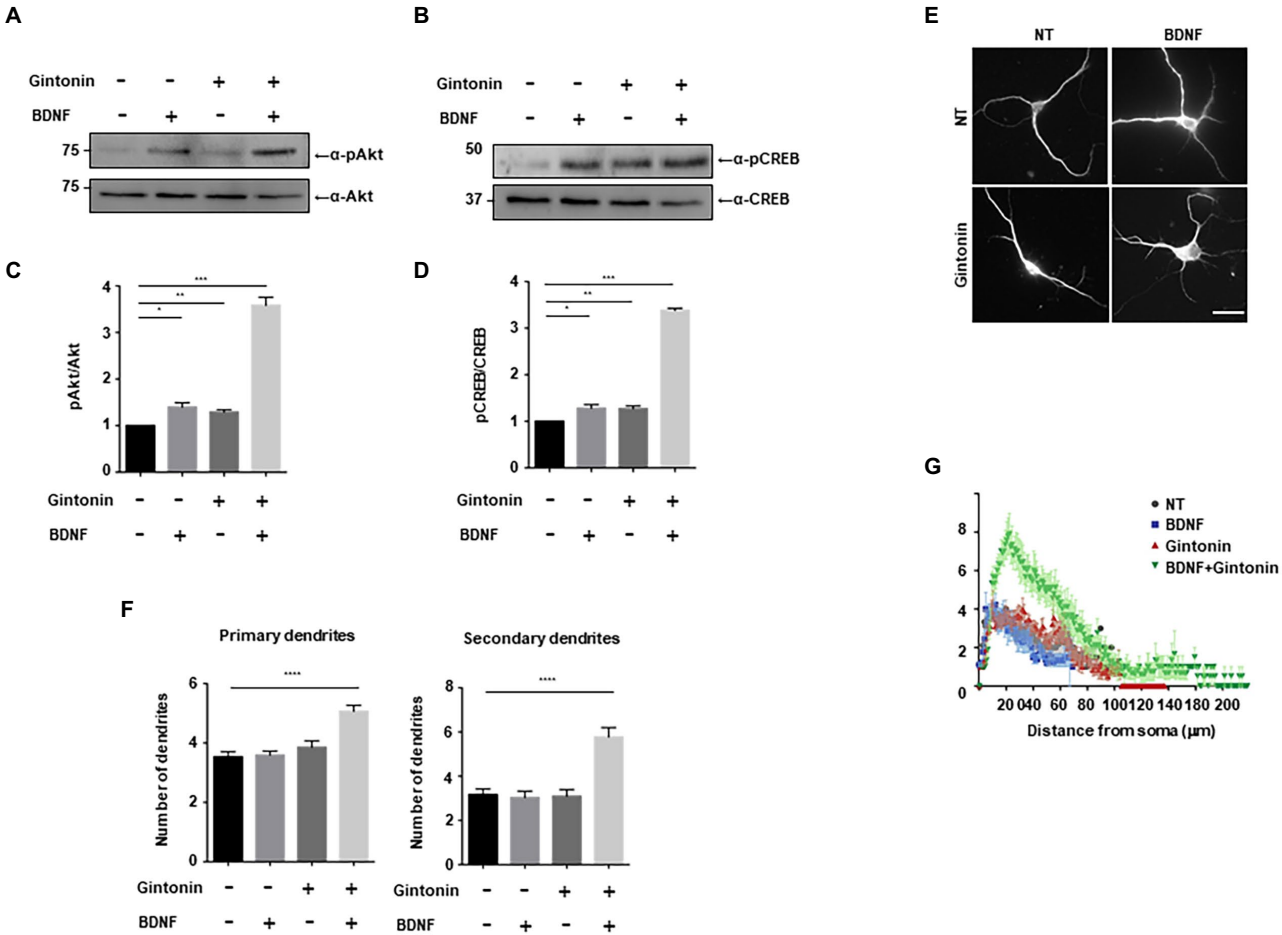
Gintonin and BDNF have a synergistic effect on dendritic growth. **(A,B)** Representative blots showing the effect of gintonin and BDNF co-treatment on Akt and CREB phosphorylation. DIV7 striatal neurons were treated with a suboptimal dose of gintonin (0.3 μg/ml) in the presence or absence of BDNF (5 ng/ml). Lysates were used for the immunoblot analysis of phospho-Akt, Akt, phospho-CREB, and CREB. **(C,D)** Densitometric quantification of the results are shown in **(A,B)**. Results are means ± SEM from three independent experiments. (*p < 0.05, **p < 0.01, ***p < 0.0005, Student’s *t*-test). **(E)** Representative images of gintonin and BDNF-treated striatal neurons. Cultured striatal neurons were treated with gintonin in the presence and absence of BDNF at DIV2. After 5 days of exposure, the cultures were fixed and stained with anti-MAP2 antibody. Neuronal processes were counted with fluorescent microscopy. Scale bar, 2 μm. **(F)** Quantitation of the number of primary and secondary dendrites is shown in **(E)**. Results are presented as means ± SEM from three independent experiments determined from the analysis of 40 neurons per condition per experiment (****p < 0.0001, Student’s *t*-test). **(G)** Sholl analysis of the dendritic arbor showing the synergistic effect of gintonin and BDNF co-treatment on striatal neurons.

BDNF, a growth factor belongs to the neurotrophin family, has a pivotal role in the preservation of dendrites in striatal and cortical neurons, and is known to be regulated by neural activity (Horch et al., 1999; Gorski et al., 2003). Dendritic abnormalities and neuronal death in the striatum can be caused by a lack of BDNF-mediated signaling (Gorski et al., 2003; Baquet et al., 2004). We investigated the effect of gintonin and BDNF on dendritic growth after verifying the effect of gintonin on the dendritic growth of striatal neurons. Gintonin and BDNF administration both cause Akt/CREB activation in striatal neurons and dendritic growth; we therefore postulated that these two stimuli would act synergistically. Gintonin at 3 μg/ml and BDNF at 50 ng/ml induced the highest level of Akt/CREB activation and dendritic growth. We performed each stimulation at a suboptimal dose to examine how gintonin and BDNF affect dendritic development. When administered with just 0.3 μg/ml of gintonin or 5 ng/ml of BDNF, a modest activation of Akt/CREB was observed. Interestingly, gintonin and BDNF co-treatment increased Akt phosphorylation by 5 times and CREB phosphorylation by 4 times, demonstrating a synergistic mode of action (Figures 6A–D). Similarly, the synergistic mode of Akt activation by gintonin and BDNF co-treatment was confirmed in cortical neurons, which are known to produce a considerable amount of BDNF (Supplementary Figure 1). Furthermore, concurrent administration of gintonin and BDNF synergistically enhanced the dendritic growth of striatal neurons in terms of the quantity of primary and secondary dendrites (Figures 6E,F). Conventional Sholl analysis confirmed that gintonin and BDNF co-treatment potentiated dendritic growth of striatal neurons (Figure 6G). These findings demonstrate that gintonin and BDNF work together and synergistically to increase the growth of striatal dendrites.

## Discussion

Dendritic growth is highly crucial as a framework for connections between neurons, which ultimately allows neurons to carry out complicated brain functions. Due to the diversity of neuronal cell types and the extrinsic signals that control them, the regulatory mechanisms of dendritic formation remain unclear. Our research demonstrated the potential of gintonin as a new molecule that stimulates dendritic growth in nerve cells. Gintonin induces the phosphorylation of Akt/CREB, which is required for dendritic growth in striatal and cortical neurons. Additionally, long-term treatment with gintonin led to the dendritic arborization of neurons and development of dendritic spines in the shape of filopodia. Gintonin stimulates dendrite development through LPA receptor signaling, indicating that the dendritogenic effect of gintonin was abolished by Ki16425, an antagonist of LPAR1/3. We further showed that gintonin successfully induced dendritic growth in striatal neurons, in which dendritic complexity was reduced by Slitrk5 and Shank3 knockdown, and could alleviate certain phenotypes related to obsessive–compulsive disorder and autism spectrum disorder. Gintonin works together with BDNF, a well-known dendritic growth factor. Gintonin did not directly stimulate TrkB, a BDNF receptor; however, at suboptimal levels, it had a synergistic effect with BDNF on Akt/CREB phosphorylation and dendritic development.

Gintonin is a molecule composed of ginseng protein and LPA, and while the function of the protein component remains unclear, it has been suggested that LPA may have biological effects *via* LPA receptors. LPA signaling is important in the development and functioning of the nervous system. LPA signaling causes migration, differentiation, apoptosis, morphological changes, and proliferation of neural network cells (Llona-Minguez et al., 2015; Yung et al., 2015). Although the role of LPA in growth cone collapse in axons is well understood, its impact on dendritic development is not well understood (Birgbauer and Chun, 2010). In this study, additional information about the role of LPA in neuronal development was provided by its impact on dendritic growth in striatal neurons. Given the molecular weight of gintonin, it induced dendritic growth and Akt/CREB activation in the nanomolar range, which was comparable to that of BDNF. Considering this activity, it may be concluded that the LPA signaling pathway significantly affects dendritic growth in striatal neurons. It would be interesting to study how LPA and BDNF signaling cooperate to support the growth, differentiation, and survival of striatal neurons.

Our study results, which found that gintonin administration improved dendritic growth defects by suppressing the expression of Slitrk5 and Shank3, have therapeutic implications. Slitrk5 and Shank3 are localized in the postsynapse of neurons and play a role in the formation, maintenance, and growth of molecular complexes that can mediate neurotransmitter signals from presynapses. Genetic analysis of patient samples and subsequent validation through animal models have established that these two genes are crucial for dendritic development and can cause obsessive–compulsive disorder and autistic spectrum disorder. These findings suggest that further research is required to confirm the therapeutic potential of gintonin and other LPAR agonists in neurological diseases caused by aberrant dendritic growth.

Overall, this study highlights novel mechanisms by which gintonin contributes significantly to the facilitation of dendritic development in CNS. These results might affect clinical efforts to use gintonins as treatments in this circumstance. According to our research, gintonin administration combined with BDNF-activated TrkB receptor activation may improve defective dendritic development, even at extremely low concentrations.

## Data availability statement

The original contributions presented in the study are included in the article/Supplementary material, further inquiries can be directed to the corresponding authors.

## Author contributions

SA and MS conceived and designed the analysis. HL, S-YN, SA, and MS developed the writing plan and drafted the manuscript. HL, KK, and YS developed the figure. All authors contributed to the article and approved the submitted version.

## Funding

This work was supported by the National Research Foundation (NRF) of Korea (NRF-2020R1A6A3A01100511 and NRF-2021R1I1A3055750), National Institute of Biological Resources (NIBR202219101), and the 2021 grant from the Korean Society of Ginseng.

## Conflict of interest

The authors declare that the research was conducted in the absence of any commercial or financial relationships that could be construed as a potential conflict of interest.

## Publisher’s note

All claims expressed in this article are solely those of the authors and do not necessarily represent those of their affiliated organizations, or those of the publisher, the editors and the reviewers. Any product that may be evaluated in this article, or claim that may be made by its manufacturer, is not guaranteed or endorsed by the publisher.

## Abbreviations

LPA: lysophosphatidic acids
LTP: long-term potentiation
PI3K: Phosphoinositide 3-kinase
PLC: Phospholipase C
IP_3_: Inositol trisphosphate
PKC: Protein kinase C
NPC: Neural precursor cells
BDNF: Brain-derived neurotrophic factor
CREB: cAMP response element-binding protein
ASD: autism-spectrum disorder
VZ: ventricular zone
OCD: obsessive–compulsive disorder
FGF, fibroblast growth factor
GDNF: glial cell line-derived neurotrophic factor
GFP: green fluorescent protein
GPCR: G-protein-coupled receptors
LPAR1: lysophosphatidic acid receptor 1
LPC: lysophosphatidic acids
TGFb: transforming growth factor beta
