# Supplementary figures and images for: Gintonin stimulates dendritic growth in striatal neurons by activating Akt and CREB
Source: Front Mol Neurosci. 2022 Oct 26;15:1014497. doi: 10.3389/fnmol.2022.1014497 (PMC9643712; doi:10.3389/fnmol.2022.1014497)

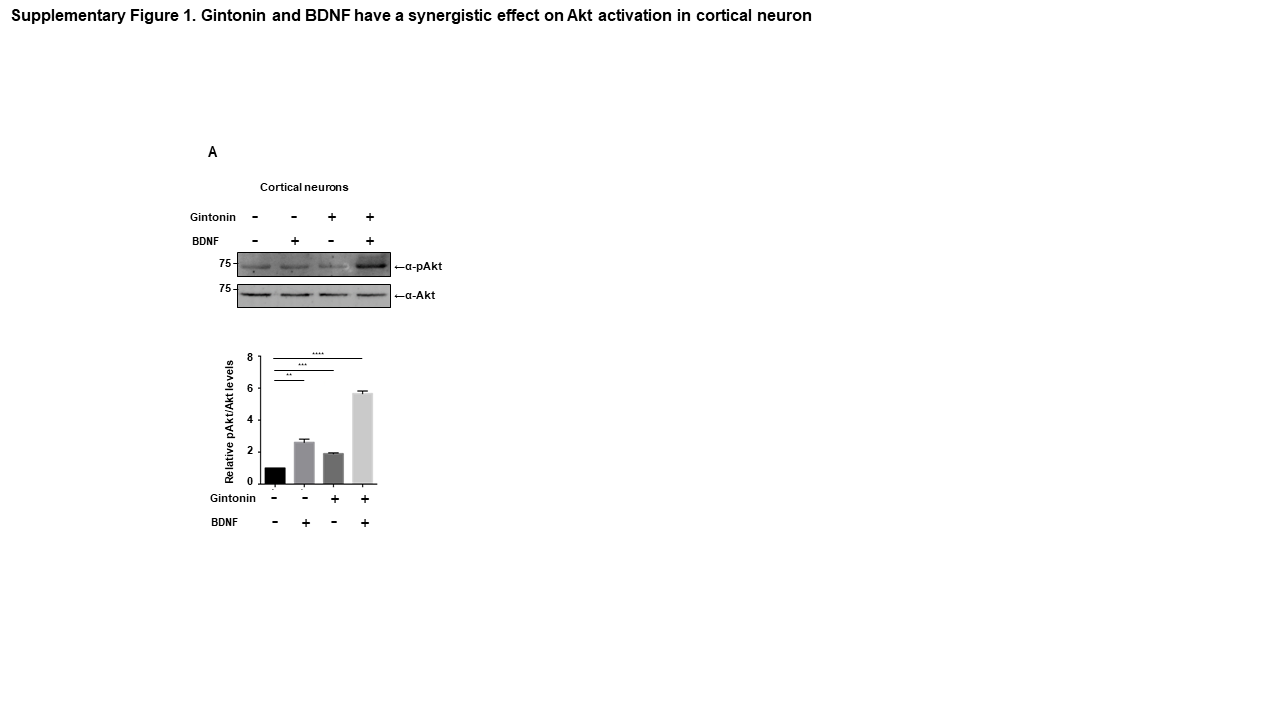

Supplement: Supplementary file 1 [file Image_1.TIF]
